# Urbanization alters communities of flying arthropods in parks and gardens of a medium-sized city

**DOI:** 10.7717/peerj.3620

**Published:** 2017-09-04

**Authors:** Edward Lagucki, Justin D. Burdine, Kevin E. McCluney

**Affiliations:** Department of Biological Sciences, Bowling Green State University, Bowling Green, OH, USA

**Keywords:** Impervious surface, Urbanization, Flying arthropods, Soil moisture, Distance to city center, Urban gardens, City parks

## Abstract

Urbanization transforms undeveloped landscapes into built environments, causing changes in communities and ecological processes. Flying arthropods play important roles in these processes as pollinators, decomposers, and predators, and can be important in structuring food webs. The goal of this study was to identify associations between urbanization and the composition of communities of flying (and floating) arthropods within gardens and parks in a medium-sized mesic city. We predicted that flying arthropod abundance and diversity would respond strongly to percent impervious surface and distance to city center, measurements of urbanization. Flying arthropods were sampled from 30 gardens and parks along an urbanization gradient in Toledo, Ohio, during July and August 2016, using elevated pan traps. A variety of potential predictor variables were also recorded at each site. We collected a total of 2,369 individuals representing nine orders. We found that flying arthropod community composition was associated with percent impervious surface and canopy cover. Overall flying arthropod abundance was negatively associated with percent impervious surface and positively associated with distance to city center. Hymenoptera (bees, wasps, ants), Lepidoptera (moths, butterflies), and Araneae (spiders) were positively associated with distance to city center. Hemiptera (true bugs), Diptera (flies), and Araneae were negatively associated with percent impervious surface. Both distance to city center and percent impervious surface are metrics of urbanization, and this study shows how these factors influence flying arthropod communities in urban gardens and city parks, including significant reductions in taxa that contain pollinators and predators important to urban agriculture and forestry. A variety of environmental factors also showed significant associations with responses (e.g. canopy cover and soil moisture), suggesting these factors may underlie or modulate the urbanization effects. More research is needed to determine mechanisms of change.

## Introduction

For the past two centuries, the global population has migrated from rural landscapes into densely populated urban environments. Currently more than half of the world’s population resides in urban regions (United Nations, 2014), and this number is growing. As more people move into urban regions, habitats are transformed into built environments and this impacts biodiversity and ecosystem processes (McKinney, 2008). The process of urbanization fragments landscapes and creates a mosaic of habitat patches of different size, use, and quality. Urbanization has been found to be a contributor to species endangerment (Czech, Krausman & Devers, 2000), and often leads to the homogenization of biotic communities (McKinney, 2006; Groffman et al., 2014). In addition, habitat loss and fragmentation in cities can alter important species interactions, such as plant–pollinator interactions (Harrison & Winfree, 2015). These changes in community structure and species interactions may affect important abiotic and biotic processes, like pollination, nutrient cycling, and decomposition (McIntyre et al., 2001), in the locations where most people now live. Thus, it is important to understand how urbanization influences organisms in order to maintain the services these organisms provide.

Urbanization can have strong positive and negative effects on a variety of organisms, making patterns of change unclear. Bird diversity, in general, is negatively affected (Blair, 2004), but total bird abundance and that of introduced species can be positively affected (Clergeau et al., 1998). Arthropod pests can have higher abundances in urban habitats, possibly due to reduced predation and parasitism (Kahn, 1988; Kahn & Cornell, 1989; McIntyre, 2000; Meineke et al., 2017), or due to direct environmental effects (Meineke et al., 2013; Dale & Frank, 2014), facilitating their proliferation and the likelihood of outbreaks. Others have argued that urbanization homogenizes biological communities because certain taxa are able to take advantage of urban environments worldwide (McKinney, 2006). But much remains to be understood about how urbanization influences biota.

Flying arthropods are abundant and diverse, and perform numerous ecosystem functions within urban environments. Many studies have shown that arthropod diversity along urbanization gradients is lowest near urban centers (Centeno, Almorza & Arnillas, 2004; Venn, Kotze & Niemela, 2003; Blair & Launer, 1997). However, one study found that ant richness can be higher with urbanization (Uno, Cotton & Philpott, 2010). Differences in abundance and richness across urban environments can result in shifts in the composition of ant assemblages (Uno, Cotton & Philpott, 2010), and bee communities (McIntyre & Hostetler, 2001; Pardee & Philpott, 2014). Some influential drivers of Hymenoptera (ants, bees, wasps) population declines in urbanized areas include habitat fragmentation and pollution (Potts et al., 2010). Studies have shown that impervious surface cover has a negative effect on specialist cavity and ground nesting bees (Geslin et al., 2016; Threlfall et al., 2015), but a positive effect on generalist honeybees (Threlfall et al., 2015). Lepidoptera (butterflies, moths) have also been shown to have reduced species richness in heavily urbanized areas (McGeoch & Chown, 1997). Much of the reduction in Lepidoptera species richness is caused by a loss of vegetation or the replacement of native with introduced plants (Majer, 1997). Furthermore, the plants many adult butterflies depend on for nectar can be more sensitive to heavy metal pollutants (Mulder et al., 2005), and this further explains the negative effects of urbanization on butterflies. Diptera abundance and community composition have also been found to vary along urbanization gradients (Avondet et al., 2003). Hemiptera abundance has been shown to increase with building cover, and to decrease with proximity to natural habitat cover in an urban environment (Philpott et al., 2014). Thus, flying arthropods may respond strongly to urbanization, but additional work is needed to help us gain a better understanding of the mechanisms behind these patterns and the potential effects on ecosystem functions and services.

Two important habitat types within urban environments are urban gardens and city parks. Urban gardens are an important source of local, healthy food (Taylor & Ard, 2015), and are increasingly used in the remediation of vacant lots in post-industrial cities like Detroit and Toledo (Our City in a Garden, 2010). Additionally, urban food production accounts for 15–20% of the global food supply (Hodgson, Campbell & Bailkey, 2011). City parks provide many social and psychological benefits to urban residents, along with environmental services like air purification and noise reduction (Chiesura, 2004). Furthermore, both urban gardens and city parks increase property values and can lead to tax revenues for cities (Luttik, 2000; Bremer, Jenkens & Kanter, 2003). Flying arthropods play important roles in urban gardens and city parks as pollinators, predators, and decomposers. Therefore, understanding how urbanization impacts flying arthropods is necessary to maintain the delivery of ecosystem services to urban gardens and city parks.

Here we examine how the abundance, diversity, and composition of flying (and floating) arthropod communities change with urbanization (percent impervious surface and distance to city center) in urban gardens and city parks. We predicted that flying arthropod abundance and diversity would be strongly correlated with percent impervious surface and distance to city center. In addition, we explored associations with other environmental variables and local habitat characteristics, in the hopes of identifying factors that might be influencing these communities across changes in urbanization, for future investigation.

## Methods

### Site location

**Figure 1:**
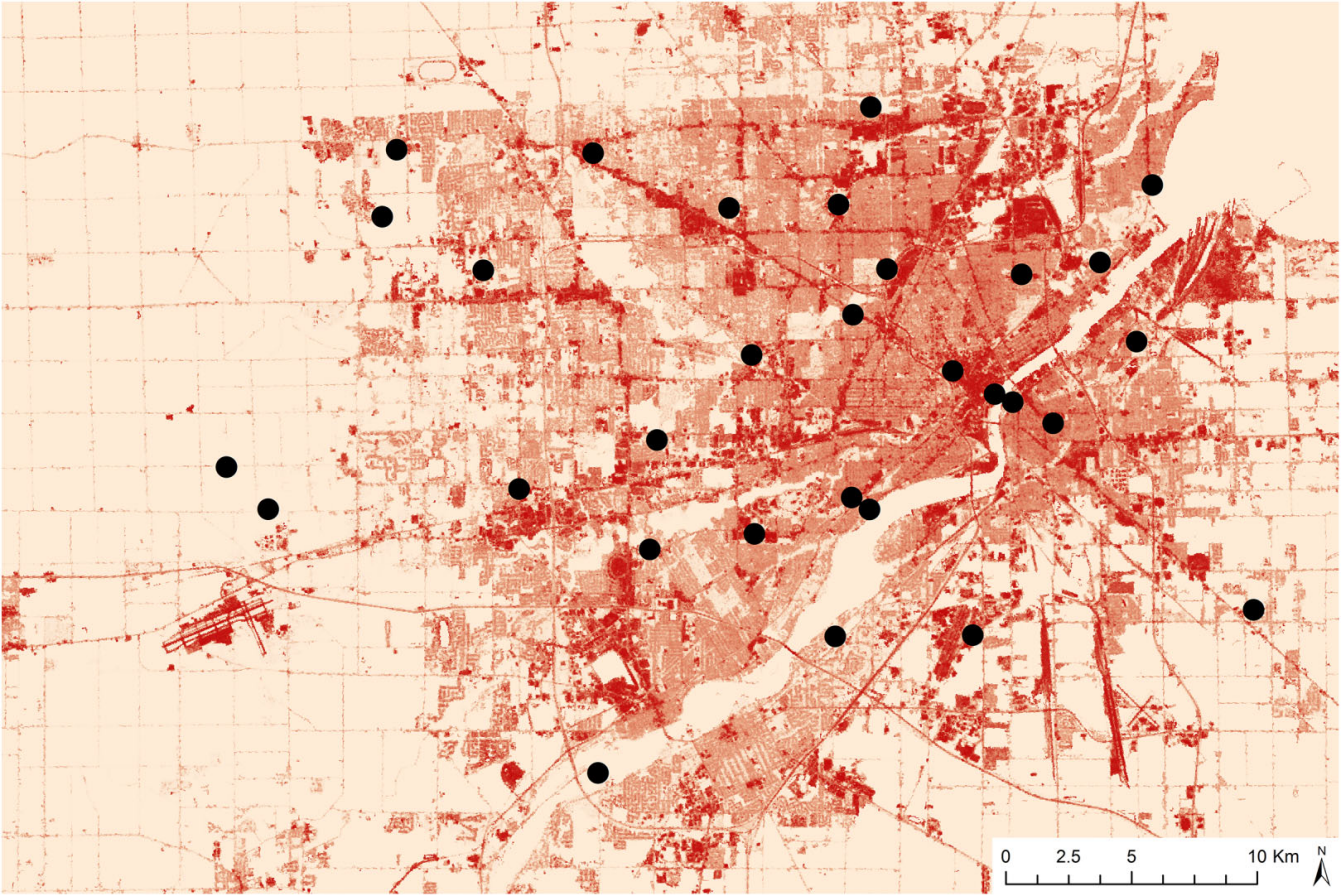
Map displaying site locations and percent impervious surface data in Toledo, Ohio. Darker colors indicate high impervious surface and light colors indicate low impervious surface.

This study was conducted in Toledo, OH, USA. We sampled flying arthropods in a total of 30 parks and garden across the metro Toledo region (Fig. 1). Sites were chosen by overlaying a grid (2 × 2 km) across a Northwest Ohio map and assigning each grid cell a number value. A random number generator was used to select which grid cells we used in our study. Within each selected grid cell, a park, or garden was chosen. Garden sites were managed by the Toledo Botanical Gardens outreach program and the MultiFaith Grows organization. Park sites were managed by the following entities: Toledo City Parks, Olander Parks Systems, Toledo Zoo, Wood County Parks, and the City of Holland.

### Sampling methods

Flying (and floating) arthropods were sampled using elevated pan traps in July and August 2016 (Permit: Ohio Division of Wildlife 17-204). Elevated pan traps were constructed by placing a 175 ml bowl atop a 1 m PVC pipe (Tuell & Isaacs, 2009). Bowls were painted white (#137990), blue (#51910), or yellow (#51806) using Krylon ColorMaster® spray paint. Each site contained three of each color type, for a total of nine elevated pan traps per site. Traps were left in the field for 24 h. Each pan trap contained a water and soap mixture. Sites were sampled once per month on days with weather conditions that were sunny with a temperature of at least 70 °F. Upon collection, insects were rinsed with water and placed into vials containing ethanol to preserve specimens. Specimens were stored and identified to order.

### Habitat characteristics

Local habitat characteristics of each site were recorded during each sampling event (Table 1). We calculated the canopy cover at the center of each site in four cardinal directions using a densiometer. We counted the total number of trees within 25 m of the site’s center. We walked a 10 m transect starting at the site’s center and counted the number of flowers and floral colors for all vegetation within 1 m on each side of the transect. Ground cover was measured by randomly placing four 1 m quadrats along each transect, calculated as a percentage in the following categories: bare ground, debris, herbaceous vegetation, leaf litter, or woody vegetation. Volumetric soil moisture was measured using a soil moisture meter (Delta-T Devices SM150) at four random points along each transect. Unshaded air temperature and relative humidity were taken with a handheld weather station (Ambient Weather WS-HT-350). Percent impervious surface was measured within a 300 m radius circle around the center of each site using the NLCD 2011 Percent Developed Imperviousness dataset from the National Land Cover Database. The distance of each site to the city center of Toledo (i.e. City Hall) was measured using Google Earth.

**Table 1:** Description of environmental variables included in this study.

Factor	Description
Percent canopy cover	Measurements of canopy cover using a densiometer in four cardinal directions of the site center
Tree counts	Total number of trees greater than 1 m in height within a 10 m radius of the site center
Flower counts	Total number of blooming flowers along a 10 m transect
Floral colors	Type of bloom color of each flower along a 10 m transect
Percent herbaceous cover	Visual estimate of herbaceous cover calculated by averaging the values from four 1 m quadrats
Percent bare ground	Visual estimate of bare ground calculated by averaging the values from four 1 m quadrats
Soil moisture	Measurement of percent soil moisture using a soil moisture meter
Temperature	Measurement of ambient temperature at the site center using a weather station
Humidity	Measurement of humidity at the site center using a weather station
Percent impervious surface	Calculated within a 300 m buffer surrounding each site center using the NLCD 2011 percent developed imperviousness dataset
Distance to city center	Distance from the site center to the center of Toledo (i.e. City Hall)

### Multivariate responses

We tested for associations between environmental factors and flying arthropod community composition with nonparametric permutational anova (*adonis*) using the “vegan” package of R (Oksanen et al., 2017). Also within this package, we used non-metric multidimensional scaling (*meta*MDS) to show differences in community composition between sites, and used the “envfit” function to show associations with environmental factors. Bray–Curtis distances were used for all community composition techniques. For these multivariate analyses, we analyzed data combined from the two months, removing the need for repeated measures statistical approaches. We used the correlation function (cor) in R to test for collinearity between environmental variables, and environmental variables were considered highly correlated at a correlation coefficient of *r* = ±0.7. When this occurred, one of the two highly correlated variables was dropped from the analysis.

### Univariate responses

All statistical analyses utilized the program R (R Development Core Team, 2015). The “vegan” package in R was used to calculate the Shannon Diversity Index and Pielou’s Evenness (Oksanen et al., 2017). We tested for associations of abundance (total flying arthropod and within order), diversity, or evenness of flying arthropods with our environmental factors and metrics of urbanization (percent impervious surface and distance to city center) using linear regression analysis. Abundance data were log-transformed, and evenness data were squared to better meet the normality and equal variance assumptions (assessed via plots of residuals). We consider α values below 0.1 to point toward potential patterns in need of further exploration and specify our exact *p* values explicitly throughout. The purpose of this research is to identify patterns rather than test hypotheses. Future research will be needed to test hypotheses and infer mechanisms.

## Results

### Collection summary statistics

We sampled and identified 2,369 individual arthropods representing nine orders (Araneae, Coleoptera, Diptera, Hemiptera, Hymenoptera, Lepidoptera, Odonata, Orthoptera, and Thysanoptera). The three most common orders in terms of relative abundance were Diptera (∼30% of all sampled insects), Hymenoptera (∼29% of all sampled insects), and Coleoptera (∼15% of all sampled insects). Diptera varied from 0 to 63 individuals per site, Coleoptera varied from 0 to 40, and Hymenoptera varied from 0 to 45.

At each site, we measured a wide range of values for our environmental variables of canopy cover (0–82.3%), number of trees (0–10 individuals), soil moisture (6.0–62.3%), impervious surface (5.6–73.3%), humidity (28.4–75%), temperature (70.3–100.4 °F), herbaceous cover (45–97.5%), bare ground (0–37.5%), distance to city center (638–21,884 m), and flower abundance (6–202). We tested for collinearity between environmental variables, and removed bare ground from further analyses due to its high collinearity with herbaceous cover (*r* = −0.78).

### Community composition results

**Figure 2:**
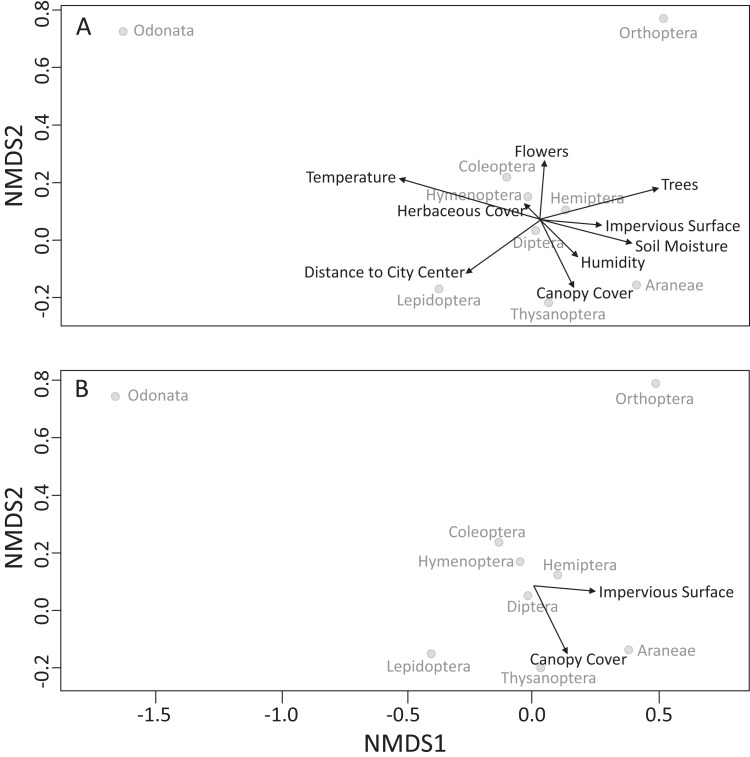
Non-metric multidimensional (NMDS) scaling analysis for flying arthropod orders sampled in Toledo, OH. (A) All environmental variables are plotted with arrows connected to each variable. (B) Impervious surface was found to have a significant association at α = 0.05 and canopy cover at α = 0.1, with flying arthropod community composition.

Our PERMANOVA (Table 2) test showed two environmental variables that were associated with flying arthropod community composition: impervious surface (*F*_1,20_ = 4.39, *p* = 0.004 at α = 0.05; Fig. 2) and canopy cover (*F*_1,20_ = 2.31, *p* = 0.057 at α = 0.1; Fig. 2).

**Table 2:** Results comparing flying arthropod community composition with environmental variables from our PERMANOVA analysis.

Source	*df*	Sum of squares	Mean of squares	*F* model	*R*^2^	*p* Value^1^
Impervious surface	1	0.38	0.38	4.39	0.13	**0.004**
Canopy	1	0.20	0.20	2.31	0.07	*0.057*
Trees	1	0.05	0.05	0.63	0.02	0.648
Soil	1	0.16	0.16	1.85	0.06	0.107
Humidity	1	0.05	0.05	0.62	0.02	0.692
Flowers	1	0.09	0.09	0.99	0.03	0.418
Temp	1	0.03	0.03	0.35	0.01	0.879
Herbaceous	1	0.15	0.15	1.78	0.05	0.113
Distance	1	0.06	0.06	0.64	0.02	0.670
Residuals	20	1.73	0.09		0.60	
Total	29	2.90			1	

**Notes:**

These results indicate that impervious surface and canopy cover were related to flying arthropod community composition (at α = 0.05 or 0.1, respectively).

1 Bold indicates significance at α = 0.05 and italics at α = 0.1.

### Distance to city center results

**Figure 3:**
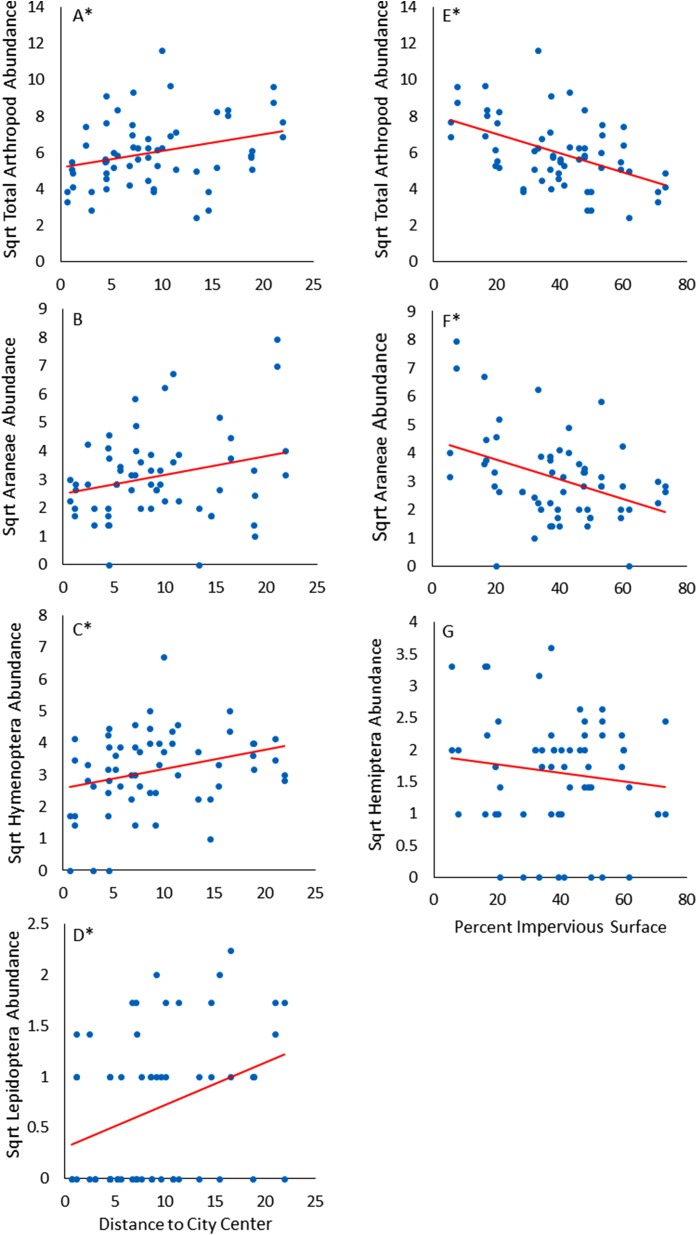
Panels displaying associations with distance to city center (A–D) and percent impervious surface (E–G). An asterisk (*) indicates significant associations at α = 0.05 while others represent significant associations at α = 0.1.

The total abundance of flying arthropods was positively associated with distance to city center (Fig. 3). For order-specific responses, we found positive associations with distance to city center for abundances of Lepidoptera (*F*_1,27_ = 10.523, *p* = 0.003), Hymenoptera (*F*_1,26_ = 4.686, *p* = 0.0398), and Araneae (*F*_1,26_ = 3.742, *p* = 0.064 at α = 0.1). Distance to city center was not associated with the diversity or evenness of flying arthropod communities.

### Percent impervious surface results

The total abundance of flying arthropods was negatively associated with impervious surface (Fig. 3). For order-specific responses, we found negative associations with percent impervious surface for abundances of Araneae (*F*_1,26_ = 4.682, *p* = 0.040), Diptera (*F*_1,28_ = 6.739, *p* = 0.0149), and Hemiptera (*F*_1,25_ = 3.228, *p* = 0.084 at α = 0.1). Percent impervious surface was not associated with the diversity or evenness of flying arthropod communities.

### Vegetation results

The total abundance of all flying arthropods combined was negatively associated with canopy cover and herbaceous cover, and positively associated with the number of flowering plants (Table 3). For order-specific response, we found negative associations with canopy cover for the abundances of Hemiptera (*F*_1,25_ = 4.385, *p* = 0.047) and Hymenoptera (*F*_1,26_ = 3.865, *p* = 0.0601 at α = 0.1). Additionally, Lepidoptera abundance was negatively associated with the number of trees (*F*_1,27_ = 4.472, *p* = 0.0438), and Hemiptera abundance was negatively associated with herbaceous cover (*F*_1,25_ = 9.664, *p* = 0.005). Vegetation factors were not associated with the diversity or evenness of flying arthropod communities.

**Table 3:** Associations between environmental factors and response factors.

Response metric	*R*^2^	Environmental factor	Relationship	*p* Value
Total arthropod abundance	0.43	Canopy cover	−	0.028*
		Impervious surface	+	0.033*
		Distance	+	0.017*
		Flowers	+	0.024*
		Herbaceous cover	−	0.005*
		Soil moisture	+	0.039*
Arthropod diversity (orders)	0.05	Soil moisture	+	0.095
Lepidoptera abundance	0.17	Trees	−	0.044*
		Distance	+	0.003*
Hemiptera abundance	0.26	Canopy cover	−	0.047*
		Impervious surface	−	0.084
		Soil moisture	+	0.039*
		Herbaceous cover	−	0.005*
Hymenoptera abundance	0.18	Distance	+	0.040*
		Canopy	−	0.060
		Soil moisture	+	0.095
Araneae abundance	0.24	Impervious surface	−	0.040*
		Distance	+	0.064
		Soil moisture	+	0.080
Diptera abundance	0.12	Impervious surface	−	0.015*

**Note:**

Multiple *R*^2^ values are given for each response metric model. An asterisk (*) indicates significant associations at α = 0.05 while others represent significance at α = 0.10.

### Soil moisture results

The total abundance of all flying arthropods was positively associated with soil moisture. Arthropod diversity was also positively associated with soil moisture. For order-specific responses, we found positive associations with soil moisture and the abundances of Hemiptera (*F*_1,25_ = 4.762, *p* = 0.039), Hymenoptera (*F*_1,26_ = 3.001, *p* = 0.0951 at α = 0.1), and Araneae (*F*_1,26_ = 3.312, *p* = 0.080 at α = 0.1).

## Discussion

Understanding how flying arthropod communities are impacted with urban gardens and city parks in urban areas is important for maintaining the many ecosystem functions flying arthropods provide. We found evidence that pollinator-containing orders of insects (i.e. Hymenoptera, Lepidoptera) are less abundant with more impervious surface and more abundant farther from the city center (i.e. Diptera). These patterns are supported across the literature for butterflies (Clark, Reed & Chew, 2007; Mauro, Dietz & Rockwood, 2007), bees (Hernandez, Frankie & Thorp, 2009), and parasitoids (Bennett & Gratton, 2012). In addition, we found evidence that orders containing both pests and predators (i.e. Araneae and Hemiptera) are less abundant with more impervious surface. These results are interesting because many of these taxa are important in providing pollination and pest control services for urban gardens and city parks. More research targeting the mechanisms of effect upon these taxa is needed.

### Associations with distance

We found more flying arthropods in general, and more Araneae, Hymenoptera, and Lepidoptera farther from the city center. Hulsmann et al. (2015) found a similar pattern with distance to city center for bumblebee abundance and diversity, while Pacheco & Vasconcelos (2007) found no effect on ant abundance in an urban region. Others have found that butterfly abundance peaks at intermediate distances (Blair & Launer 1997). Peaks in abundance at intermediate distances may be explained by additional food and water resources made available in suburban regions, while peaks at distances further from the urban core are often explained by plant community composition and density (Hulsmann et al., 2015).

### Associations with impervious surface

We found that impervious surface was associated with shifts in flying arthropod community composition, with fewer flying arthropods overall with higher impervious surface. In addition, Hemiptera, Araneae, and Diptera showed lower abundances with more impervious surface. Studies have shown similar patterns for bumblebees (Ahrne, Bengtsson & Elmqvist, 2009), ground spiders (Kaltsas et al., 2014), and tree spiders (Meineke et al., 2017). However, scale insects (Hemiptera) are positively affected by impervious surface (Dale, Youngsteadt & Frank, 2016; Speight et al., 1998). Other studies have found percent impervious surface to have no effect on the abundance of arthropods (Pacheco & Vasconcelos, 2007). One mechanism to explain why impervious surface reduced arthropod abundance is a species-area effect, since impervious surfaces can lead to a loss in habitat area (McKinney, 2008). Another mechanism is a physiological effect of impervious surface on arthropods. Diamond et al. (2017) found difference in physiological limits for ants sampled at sites with high and low impervious surface. However, many other possibilities exist (e.g. increased soil contaminates, reduced nesting sites).

It is interesting to note that except for spiders, the orders influenced by distance to city center were different than those influenced by percent impervious surface. This suggests that Hymenoptera and Lepidoptera may be more influenced by habitat fragmentation and a loss of connectivity, while Hemiptera and Diptera may be more influenced by local habitat characteristics associated with impervious surface (e.g. increased temperatures). This hypothesis warrants further testing.

### Association with vegetation factors

We found negative associations with canopy and herbaceous cover on Hymenoptera and Hemiptera abundance, as well as overall flying arthropod abundance. Additionally, canopy cover was associated with the composition of flying arthropod communities. Previous studies in urban systems support our findings on canopy cover, but not herbaceous cover. Studies show that canopy cover reduces herbivorous ground arthropod abundance (Philpott et al., 2014), and has a significant impact on ant community composition (Uno, Cotton & Philpott, 2010). But these studies found herbaceous cover to have positive or no effects on arthropods, and others have found similar positive effects of herbaceous cover on arthropods (Pinna et al., 2008). The differences between our findings (negative associations with herbaceous cover) and those of others (positive or no associations) may be due to the herbaceous cover structure or composition (i.e. height, diversity, or type). Studies have found that vegetation height is an important predictor of community composition for leafhoppers and grasshoppers (Strauss & Biedermann, 2006). Our findings that the total arthropod abundance was negatively associated with herbaceous cover, but positively associated with flowing plants, could be explained by aspects of herbaceous cover for which we did not account. One might expect flowering plants to be associated with herbaceous vegetation in undeveloped areas, but we suggest that this relationship may not hold in managed urban landscapes, where turf grass is part of the herbaceous cover. Additionally, a previous study showed that Hymenoptera were more attracted to specific plant species, and not necessarily diverse gardens (Barbir et al., 2015). Combined this suggests that the relative abundance of herbaceous vegetation should not necessarily be expected to be positively associated with arthropod abundance in urban areas.

### Associations with soil moisture

Soil moisture also had strong associations with flying arthropod abundance. Soil moisture was the only factor to have positive associations on arthropod diversity, and it was positively associated with the abundance of Araneae, Hemiptera, and Hymenoptera. Studies have found positive effects of soil moisture on arthropod movement (Green, Scharf & Bennett, 2005), arthropod water content (McCluney, Burdine & Frank, 2017), and arthropod abundance (Allen et al., 2014), but research is lacking on the role of soil moisture in altering community composition and diversity. However, a study found that the absolute number of insect species increased with increasing soil moisture levels and suggests that soil moisture plays a key role in overall ecosystem health (Janzen & Schoener, 1968). Our finding that soil moisture is associated with flying arthropod abundance and diversity is interesting because urban gardens (and many city parks) are irrigated and receive water inputs. Studies have shown that irrigation can positively impact arthropod abundance (Cook & Faeth, 2006), and these inputs could be important in maintaining abundant and diverse flying arthropod communities in urbanized sites.

### Conclusion

Understanding drivers of flying arthropod declines is necessary in maintaining the important services they provide in urban gardens and city parks. Upwards of 150 agricultural crops in the US require pollination services, and flying arthropods are the primary pollinator of these crops. Additionally, pest control services are important in reducing crop loss. With estimates that 15–20% of the world’s food supply comes from urban agriculture (Maxwell et al., 2000), conservation of flying arthropods with urban environments should be an issue of global concern. The patterns we observed indicate that urbanization plays an important role in shaping arthropod communities, and particularly may reduce the abundance of Lepidoptera and Hymenoptera.

## Supplemental Information

10.7717/peerj.3620/supp-1 Supplemental Information 1 Code for data analysis in R. The R code used to analyze our data in the statistical program R.

10.7717/peerj.3620/supp-2 Supplemental Information 2 Raw data for data analysis using R. Raw dataset associated with the R code that was used to run all anova and regression analyses.

10.7717/peerj.3620/supp-3 Supplemental Information 3 Raw data for NMDS analysis (predictors). The first file needed to run the NMDS analysis (predictors).

10.7717/peerj.3620/supp-4 Supplemental Information 4 Raw data needed for NMDS analysis (orders). The second file needed to run the NMDS analysis (orders). The file contains total counts of all individual flying arthropods.

## Additional Information and Declarations

### Field Study Permissions

The following information was supplied relating to field study approvals (i.e. approving body and any reference numbers): Field experiments were approved by the Ohio Division of Wildlife (Wild Animal Permit: 17-204).

### Data Availability

The following information was supplied regarding data availability: Burdine, Justin (2017): 2016 Arthropod Data. figshare. DOI 10.6084/m9.figshare.4776634.v1
