# Supplementary material for: Urbanization alters communities of flying arthropods in parks and gardens of a medium-sized city
Source: PeerJ. 2017 Sep 4;5:e3620. doi: 10.7717/peerj.3620 (PMC5590548; doi:10.7717/peerj.3620)
Supplement: Supplemental Information 1 — The R code used to analyze our data in the statistical program R. [file peerj-05-3620-s001.docx]

# NMDS Analysis

library(vegan)

orders=read.csv(file="Total_Orders.csv")

predictors=read.csv(file="Average_Predictors.csv")

all=read.csv(file="All_Time.csv")

summary(predictors)

nmds1<-metaMDS(orders,distance="bray",trace=FALSE)

nmds1

names1<-as.character(predictors$Class)

names1

#Display names of orders and sites

pl<-plot(nmds1,type="none")

points(nmds1,display="sp",col="dark gray",pch=20,cex=0.8)

text(nmds1,display="sp",col="black",pch=20,cex=0.8) #OR use the identity function

identify(pl, "species", col="darkgray",cex = 0.8)

#envfit fits an environmental variables onto an ordiation

fit<-envfit(nmds1~Canopy+Trees+Soil+Humidity+Flowers+Temp+Imp+Herb+Distance,predictors,perm=4999,col="dark gray")

plot(fit, col="black")

#adonis - PERMANOVA using distance matrices

stat.test<-adonis(orders~Imp+Trees+Canopy+Soil+Flowers+Humidity+Distance+Herb,predictors,permutations=2000,method="bray")

stat.test

# Fit of significant environmental variables

pl<-plot(nmds1,type="none")

text(nmds1,display="sp",col="black",pch=20,cex=0.8) #OR use the idenity function

fit<-envfit(nmds1~Canopy+Imp,predictors,perm=4999)

plot(fit, col="black")

#Calculating Diversity and Evenness

diversity(orders,index="shannon")

H<-diversity(orders) #Shannon Diversity Index

J<-H/log(specnumber(orders)) # Pielou's Evenness

all$Diversity<-c(H) #adds diversity to the predictors dataframe

all$Evenness<-c(J) #adds evenness to the predictors dataframe

#Univariate Responses

lm1=lm(Sq.Abundance~Distance+Canopy+Soil+Flowers+Imp+Herb,data=all)

lm2=lm(Diversity~Soil,data=all)

#Coleoptera

lm.coleoptera=aov(Coleoptera~Distance+Canopy+Trees+Soil+Humidity+Flowers+Temp+Imp+Herb+Error(Site), data=all)

summary(lm.coleoptera)

#Lepidoptera

lm.lepidoptera=aov(Sqrt.Lepidoptera~Distance+Trees+Error(Site), data=all)

summary(lm.lepidoptera)

#Hymenoptera

lm.hymenoptera=aov(Sqrt.Hymenoptera~Distance+Canopy+Soil+Error(Site), data=all)

summary(lm.hymenoptera)

#Araneae *

lm.araneae=aov(Sqrt.Araneae~Distance+Soil+Imp+Error(Site), data=all)

summary(lm.araneae)

#Diptera *

lm.diptera=aov(Log.Diptera~Imp+Error(Site), data=all)

summary(lm.diptera)

plot(a.diptera)

#Hemiptera *

a.hemiptera=aov(Sqrt.Hemiptera~Class+Error(Site), data=all)

summary(a.hemiptera)

lm.hemiptera=aov(Hemiptera~Imp+Canopy+Soil+Imp+Herb+Error(Site), data=all)
